# Effects of volatile anesthetics on peripheral nerve regeneration in a sciatic cut repair murine model

**DOI:** 10.3389/fncel.2026.1831381

**Published:** 2026-06-19

**Authors:** Yameng Xu, Ying Yan, Wilson Z. Ray, Umeshkumar Athiraman

**Affiliations:** 1 The Institute of Materials Science and Engineering, Washington University, St. Louis, MO, United States; 2 Department of Neurological Surgery, Washington University, St. Louis MO, United States; 3 Department of Orthopedic Surgery, Washington University, St. Louis, MO, United States; 4 Department of Biomedical Engineering, Washington University, St. Louis, MO, United States; 5 Department of Anesthesiology, Washington University, St. Louis, MO, United States

**Keywords:** axonal regrowth, functional outcomes, myelin regeneration, peripheral nerve injury, volatile anesthetics

## Abstract

**Background:**

We previously showed that 2% isoflurane conditioning promoted axonal and myelin regeneration leading to improved functional outcomes after peripheral nerve injury (PNI) in a sciatic nerve cut repair model. Whether the 1 MAC (minimum alveolar concentration) dose of isoflurane (1.2%) and other commonly used volatile anesthetics such as sevoflurane (2%), and desflurane (6%), support peripheral nerve regeneration is not known. The aim of the current study is to examine these possibilities in a rodent sciatic nerve injury model.

**Methods:**

Twelve-week-old male Lewis rats underwent sciatic nerve cut and repair and were divided into following groups. (1) control (no treatment), (2) isoflurane (1.2%), (3) sevoflurane (2%), and (4) desflurane (6%). Volatile anesthetic conditioning was achieved by administering isoflurane, sevoflurane, and desflurane for 1 h, beginning 1-h post sciatic nerve cut and repair, and the anesthetic exposure was repeated for the next two consecutive days for 1 h. Functional outcomes such as compound muscle action potential (CMAP), evoked muscle force (tetanic and specific tetanic force), wet muscle mass, and axonal counts were measured at 12 weeks post-surgery.

**Results:**

A significant increase in the axonal numbers and myelin width was observed in the desflurane group. This was associated with the improvement in the specific tetanic force measured at 12 weeks post-surgery.

**Conclusion:**

Desflurane promoted axonal regeneration and myelination at 12 weeks post-peripheral nerve injury and repair in a murine model. Future experiments focusing on identifying the optimal dose to improve functional outcomes, and the molecular mechanisms underlying desflurane’s potential protective effect are warranted.

## Introduction

Peripheral Nerve injuries (PNI) constitute approximately 560,000 cases per year in the United States alone (Brattain, 2012). PNI leads to a partial or complete loss of sensory/motor functions resulting in long-term disruption of patient lives. On top of that, traumatic nerve injuries more commonly affect younger patients leading to significant socioeconomic burdens (Ciaramitaro et al., 2010; Jaquet et al., 2001; Lee et al., 2014; Taylor et al., 2008). Despite several advancements in the field to improve functional outcomes after PNI, current treatments remain suboptimal, necessitating the development of new therapeutic interventions (Grinsell and Keating, 2014; Lopes et al., 2022).

Previously we have demonstrated that brief exposure to an anesthetic conditioning stimulus (isoflurane 2% for 1 h), initiated 1 h after the sciatic nerve cut repair in a murine model and repeated for next 2 or 5 days (for a total of 3 or 6 days) promoted myelin and axonal regeneration leading to markedly improved evoked muscle force at 12 weeks post PNI and repair (Xu et al., 2024). These results suggested a neuroprotective role for isoflurane conditioning in improving functional outcomes after PNI. Though the dose of isoflurane (2%) used in our previous study has been frequently applied in several preclinical studies, it is not the usual dose applied clinically to anesthetize the patients (Nickalls and Mapleson, 2003). In addition, it is important to know the potential of other commonly used volatile anesthetics (sevoflurane/desflurane) on the peripheral nerve regeneration after PNI. Understanding the differential impact of various anesthetics will allow us to identify the ideal anesthetic which could ultimately improve patient outcomes after PNI. Our current study is designed to examine the impact of 1 MAC (minimum alveolar concentration - The concentration at which 50% of patients do not move in response to a surgical incision) dose of commonly used volatile anesthetics such as isoflurane, sevoflurane, and desflurane on the peripheral nerve regeneration in a sciatic cut and repair murine model. Our hypothesis is that volatile anesthetics will promote axonal and myelin regeneration leading to improved functional outcomes after peripheral nerve injury and repair.

## Materials and methods

**FIGURE 1:**
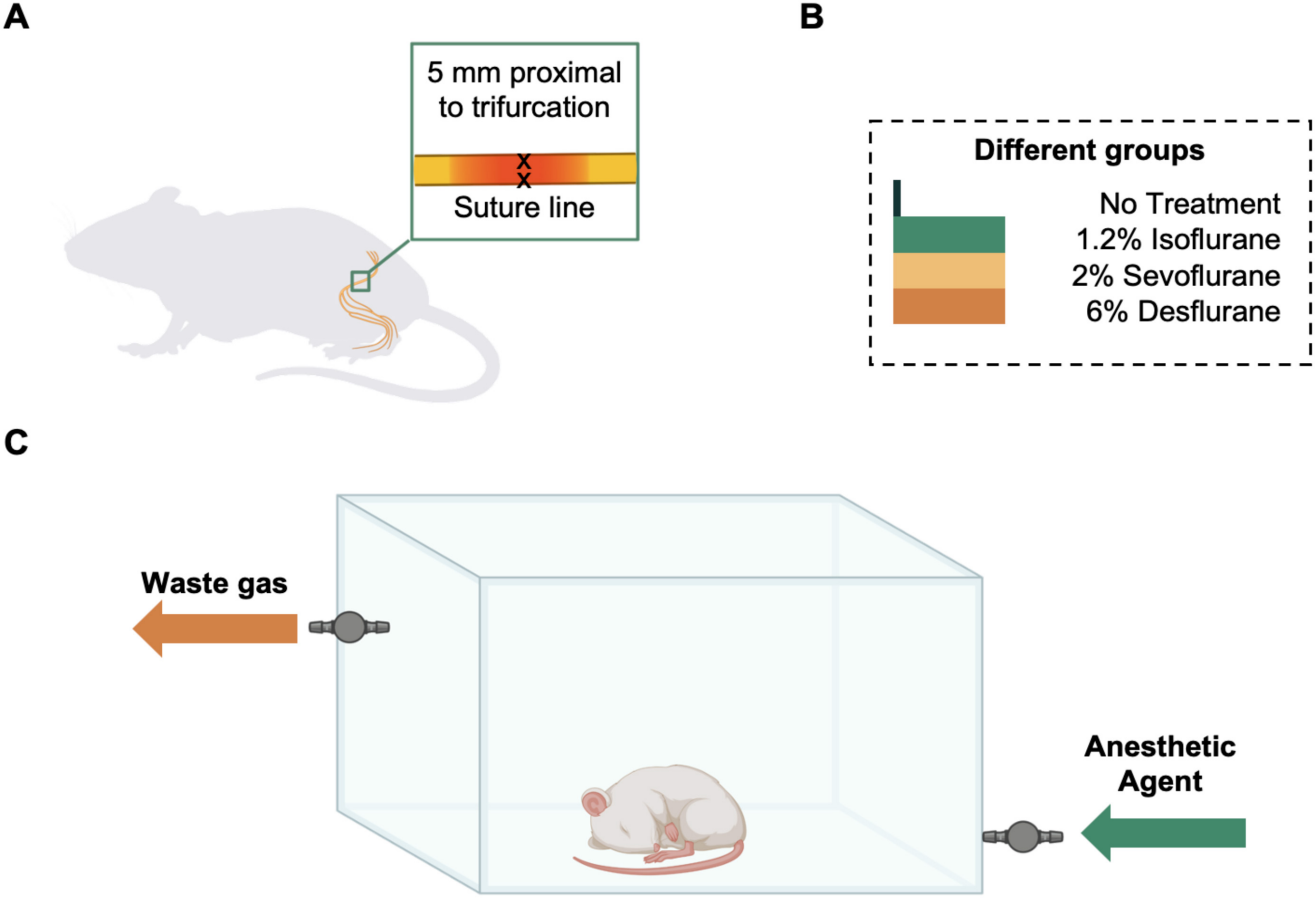
Diagram illustrations of study design. **(A)** Schematic diagram of the sciatic cut repair in rats. **(B,C)** Schematic diagram of volatile anesthetics exposure (Isoflurane 1.2%, Sevoflurane 2%, Desflurane 6%). Created with BioRender.com.

The Washington University in Saint Louis animal care and use committee approved all the experiments conducted in this study (Protocol numbers, 21-0192 and 24-0401). Thirty-two adult male Lewis rats weighing 250–275 g, were obtained from Charles River Laboratories (Wilmington, MA) for the experiments. All animals were housed in a central animal care facility and provided with food and water ad libitum. Eight rats were randomized into each of the four groups (Table 1). Group I is the control group with no anesthetic exposure and represented as no treatment group, and groups II, III, and IV were exposed to 1 MAC dose of commonly used volatile anesthetics such as isoflurane (1.2%), sevoflurane (2%), and desflurane (6%), respectively. Twelve weeks post-surgery, functional recovery was evaluated by recording compound muscle action potentials (CMAPs) from the *tibialis anterior* (TA) muscle, evoked muscle force measurements from the *extensor digitorum longus* (EDL) muscle, and muscle mass of the EDL. Immediate post functional recovery assessment, the sciatic nerves were harvested to analyze the axonal counts and microstructure of axons. The overall design of the study is represented in Figure 1.

**TABLE 1:** Study design.

Group	Group ID	Group size	Surgery model	Treatment
I	Control	*N* = 8	Transection + repair	No treatment
II	Isoflurane	*N* = 8	Transection + repair	1.2% isoflurane for 1 h, 1-h post-injury (3 days)
III	Sevoflurane	*N* = 8	Transection + repair	2% sevoflurane for 1 h, 1-h post-injury (3 days)
IV	Desflurane	*N* = 8	Transection + repair	6% desflurane for 1 h, 1-h post-injury (3 days)

### Sciatic nerve cut repair model

A single dose of Buprenorphine SR (1.2 mg/kg, subcutaneous) was administered 1 h before surgery to alleviate postoperative pain and discomfort. Anesthetic induction and maintenance were achieved by 4% and then by 2% isoflurane, respectively for the sciatic cut repair surgery and for the end point assessments. Under aseptic conditions, the right gluteal muscle was incised and bluntly dissected to expose the right sciatic nerve with careful isolation from the surrounding connective tissue. The isolated sciatic nerve is then sharply transected at 5 mm proximal to the sciatic trifurcation followed by realignment in an end-to-end fashion using 3–4 stitches of 9–0 nylon suture under microscopic guidance to ensure minimal tension. The muscle and skin were then closed using 5–0 polyglactin and 4–0 nylon sutures, respectively. Post-surgery, 5 ml of warm sterile saline was administered to the rats subcutaneously to avoid potential dehydration and the animals were then moved to a recovery area located in the surgical suite. When animals were fully awake and moving around, they were moved back to the animal facility. All animals were monitored postoperatively for signs of infection and distress (including weight loss, autophagy, lack of grooming, and wound complications) for a total of 7 days (twice daily for 3 days, and once for 4 days).

### Anesthetic treatment

A custom-made flexi glass chamber (Figure 1) was used for volatile anesthetic exposure. Animals in group II, III, and IV were exposed to 1 h of isoflurane (1.2%), sevoflurane (2%), or desflurane (6%) combined with medical air (oxygen 21%, Nitrogen 78%), 1-h post sciatic cut repair surgery. The anesthetic exposure was repeated for the next two consecutive days, so each animal received a total of three doses of anesthetics. The MAC of commonly used volatile anesthetics (isoflurane, sevoflurane, desflurane) differs between rodents and humans, and it also varies between different rat strains (Gong et al., 1998; Orliaguet et al., 2001). The concentrations of volatile anesthetics used in the current experiments were based on the standard anesthetic doses in the human patients (Nickalls and Mapleson, 2003). The three-time exposure for all three volatile anesthetics in our current experiment is based on our previously published article (Xu et al., 2024), where we noticed that the three or six-time isoflurane exposures provided meaningful improvement in functional outcomes and no statistically significant difference was found between those two exposures.

### Functional assessment

Compound muscle action potentials of the reinnervated TA muscle and the evoked muscle forces of the reinnervated EDL muscle were used to evaluate sciatic nerve regeneration at 12 weeks post-surgery as described previously (Xu et al., 2024). All functional assessments were performed under room temperature.

### Compound muscle action potentials (CMAPs)

The sciatic nerve on the operated side was exposed, and the CMAPs from the reinnervated TA muscle was measured at the proximal sciatic nerve by an automated functional assessment station (FASt System, Red Rock Laboratories, St. Louis, MO). The stimulation electrodes were wrapped around the sciatic nerve about 5 mm proximal to the surgical site. The recording electrodes were inserted into the belly of the TA muscle (4–5 cm to the stimulation electrodes), while the reference electrode was placed at the base of the tail. A pulse stimulation (1 mA, 0 Hz) was applied using a 2-channel microelectrode amplifier (Model 1800 A-M Systems, Sequim, WA), and the CMAP signal was amplified 100x to measure the maximum values of the CMAPs.

### Evoked muscle force

Evoked muscle force was measured after the CMAP assessment. Briefly, the distal tendon of the EDL was cut and fixed to a 10N load cell (S100, Strain Measurement Devices, Wallingford, CT) and the twitch contractions were measured using a single pulse along with determining the optimal muscle length for isometric force generation by the EDL muscle. Tetanic muscle forces were then measured by increasing the stimulation frequency steadily from 80 to 200 Hz. The maximum twitch forces and tetanic forces (F_*o*_) were calculated using the FASt System and recorded manually. The specific tetanic muscle force (SF_*o*_) was obtained from tetanic forces (F_*o*_) and physiological cross-sectional area (PCSA) of the EDL muscle by the formula


$$S\,F_{o}=F_{o}/P\,S\,C\,A,$$

where the PSCA was calculated by


$$P\,S\,C\,A=\left(M\times \cos\,\theta \right)/\left(\rho \times L_{o}\times \frac{L_{f}}{L_{m}}\right)$$

In these equations, M represents the muscle mass (weighed as described below), *cos*θ represents the angle of pennation of the EDL muscle, ρ represents the density of mammalian skeletal muscle, L_o_ represents the optimal muscle length, and L_f_/L_m_ represents the ratio of fiber length to muscle length in rat EDL muscle (Moore et al., 2011).

### EDL muscle atrophy quantification

Post functional end point assessments, animals were euthanized by the intracardiac administration of sodium pentobarbital 190 mg/kg followed by confirmation with absent heartbeat and respiration. EDL muscles were then harvested from operated and non-operated sides for the quantification of the muscle mass. The relative degree of muscle atrophy was then measured using the wet muscle mass ratio between the operated and the non-operated side.

### Histological analysis

The sciatic nerves on the operated side were fixed by 3% glutaraldehyde in 0.1 M phosphate solution (PS) for at least 24 h at 4 °C, followed by overnight osmication and dehydration with 1% osmium tetroxide (Fisher Scientific, Waltham, MA) and gradient ethanol/propylene oxide solutions (Sigma-Aldrich, St. Louis, MO), respectively. The nerves were then embedded into epoxy blocks (Araldite(R) M/hardener DDSA, Sigma-Aldrich, St. Louis, MO) and the nerve embeddings of 5-mm distal to cut/repair spot were sectioned to several-micron sections, followed by staining with 0.5% toluidine blue (Sigma-Aldrich, St. Louis, MO) for 90 s. The axonal structure and the numbers were then examined under different magnifications (× 40, × 100, and × 1,000) using Clemex software (Clemex, Fenton, MI).

### Statistical analysis

Origin v8.5 (Origin, Northampton, MA) and GraphPad Prism 8 (GraphPad Software, Boston, MA) were used for statistical analysis. Data are presented as mean ± SD. Brown-Forsythe and Kolmogorov–Smirnov test were used to examine the homogeneity of variances and the normality of the data. To identify differences between groups, one-way ANOVA followed by Tukey’s HSD multiple comparison test was applied for normally distributed data with equal standard deviations, Brown-Forsythe and Welch ANOVA test followed by Dunnett’s T3 multiple comparisons test for normally distributed data sets with unequal standard deviations, and Kruskal–Wallis followed by Dunn’s multiple comparison test for the non-normally distributed data. Statistical significance was set at *P* < 0.05.

## Results:

### Compound muscle action potential (CMAP)

**FIGURE 2:**
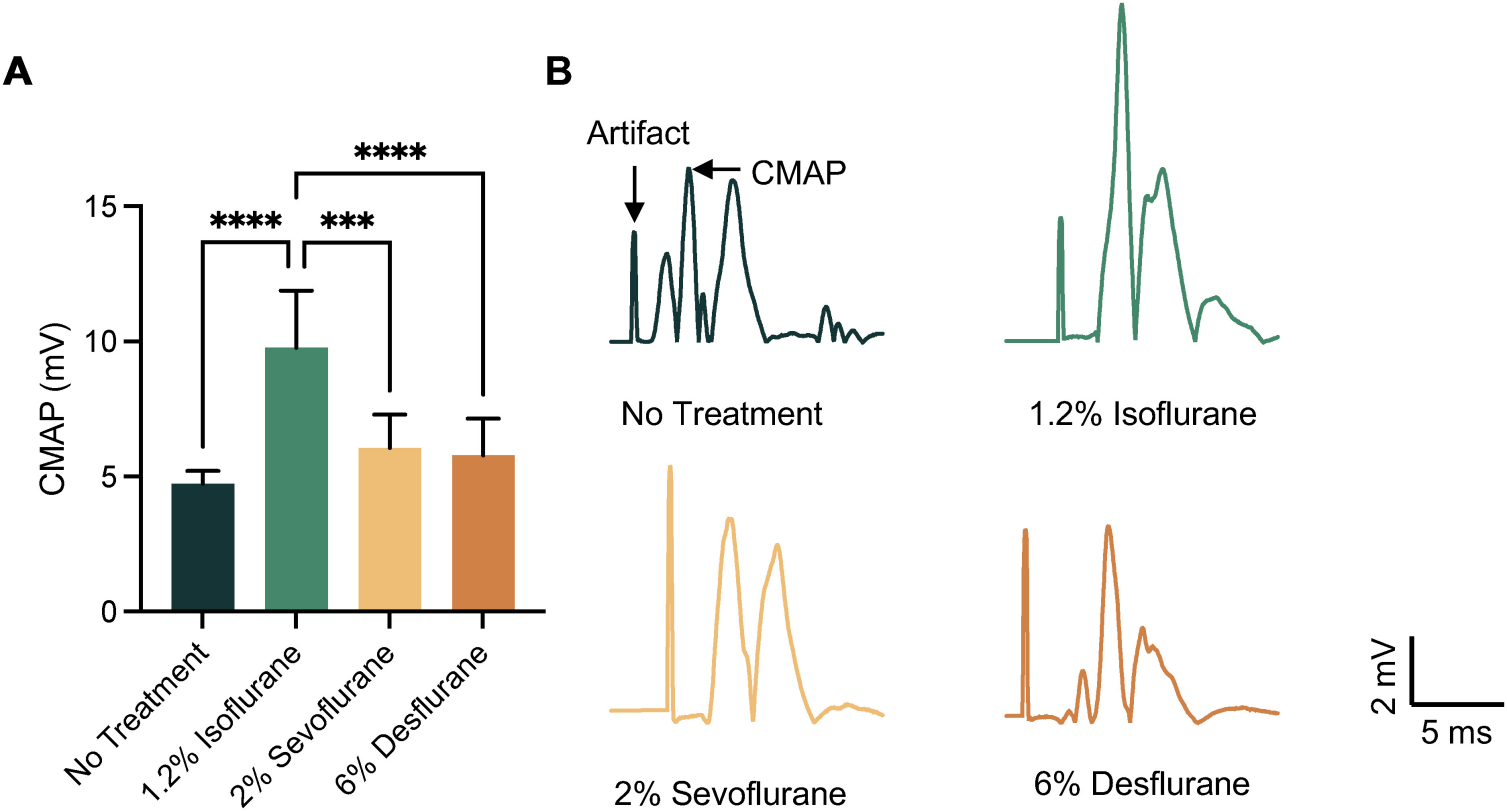
Compound muscle action potentials (CMAPs) after anesthetic treatment. Amplitude of CMAPs evoked in the tibialis anterior (TA) muscle following 0 Hz stimulation of the sciatic nerve at 12 weeks post-surgery. Data are represented as mean ± SD. **(A)** CMAPs, *p* < 0.05, Isoflurane vs control (No treatment), Sevoflurane, Desflurane by ANOVA with Tukey multiple comparisons test. **(B)** Representative CMAP curves of each group. *N* = 8 individual animals per group. Dark blue, no treatment; Green, isoflurane; Yellow, sevoflurane; Orange, desflurane. ***p < 0.001; ****p < 0.0001.

Figure 2 shows the CMAPs for all the groups measured at 12 weeks post-surgery. Isoflurane (9.77 ± 2.11 mV) showed a significant improvement in the muscle action potentials compared to the control group (4.73 ± 0.48 mV), and other anesthetic groups (sevoflurane, 6.05 ± 1.22 mV), (desflurane, 5.79 ± 1.36 mV). A non-significant trend in improvement was observed between the sevoflurane and desflurane groups compared to the control group.

### Evoked muscle force

**FIGURE 3:**
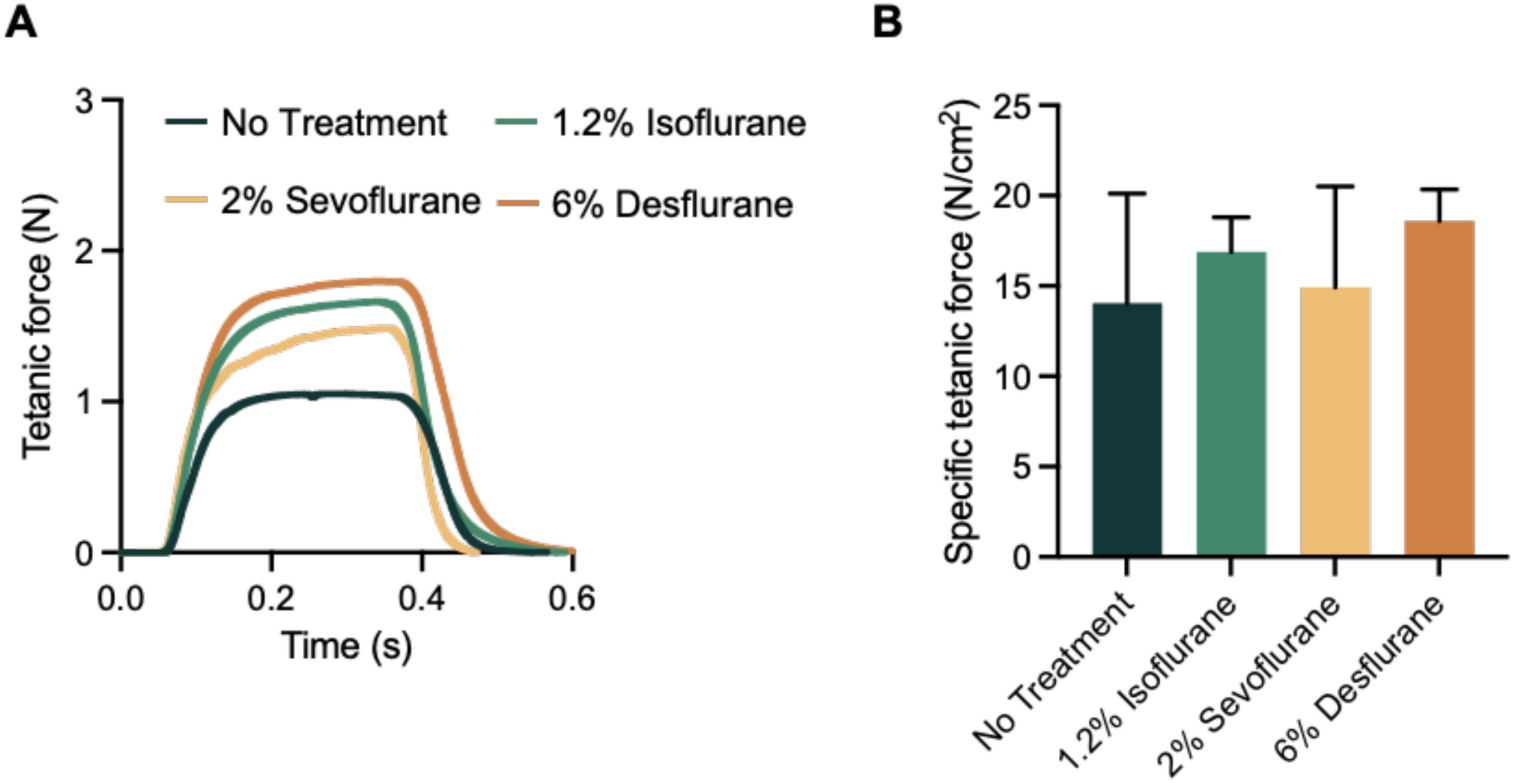
Evoked tetanic muscle forces after anesthetic treatment. Evoked muscle tetanic forces measured from extensor digitorum longus (EDL) after stimulation of the sciatic nerve at 12 weeks post-surgery. Data are represented as mean ± SD. **(A)** Representative curves of tetanic forces for each group. **(B)** Specific tetanic force, *p* > 0.05, control (No treatment) vs. Isoflurane, Sevoflurane, Desflurane by Kruskal-Wallis test with Dunn’s multiple comparisons test. *N* = 8 individual animals per group. Dark blue, no treatment; Green, isoflurane; Yellow, sevoflurane; Orange, desflurane.

Figure 3 shows the tetanic force, and specific tetanic force of EDL muscle measured at 12 weeks post-surgery. Though no statistically significant differences were noticed among the groups, the animals treated with isoflurane (1.68 ± 0.17 N; 16.88 ± 1.92 N/cm^2^), and desflurane (1.80 ± 0.13 N; 18.59 ± 1.76 N/cm^2^), showed a trend in improved muscle force (tetanic force; specific tetanic force) compared to the control group (1.29 ± 0.64 N; 14.05 ± 6.06 N/cm^2^), whereas the animals treated with sevoflurane (1.38 ± 0.53 N; 14.93 ± 5.59 N/cm^2^), showed a muscle force similar to the control group.

### Muscle mass

**FIGURE 4:**
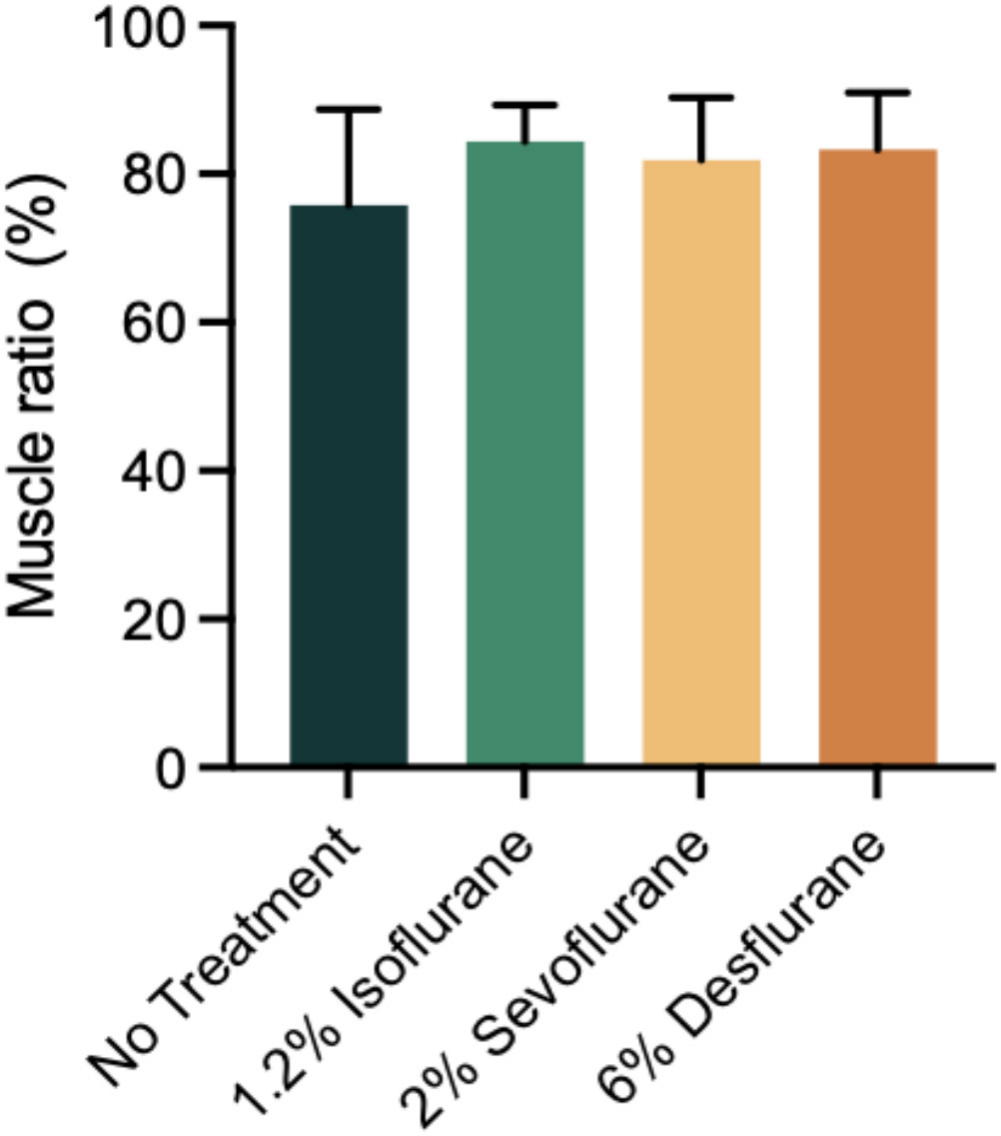
Muscle mass after anesthetic treatment. Wet muscle mass ratio measured from extensor digitorum longus (EDL) from both the operated and unoperated side at 12 weeks post-surgery. Data are represented as mean ± SD. *p* > 0.05, control (No treatment) vs. Isoflurane, Sevoflurane, Desflurane by ANOVA with Tukey multiple comparisons test. *N* = 8 individual animals per group. Dark blue, no treatment; Green, isoflurane; Yellow, sevoflurane; Orange, desflurane.

Figure 4 shows the wet muscle mass ratio measured in the EDL muscle. The muscle mass ratio was higher in all the anesthetic treatment groups (Isoflurane, 84.41 ± 4.91%; Sevoflurane, 81.86 ± 8.42%; Desflurane, 83.27 ± 7.71%) compared to the control group (75.77 ± 12.92%), though no marked difference was noticed among the groups.

### Histomorphometric analysis of axonal regeneration

**FIGURE 5:**
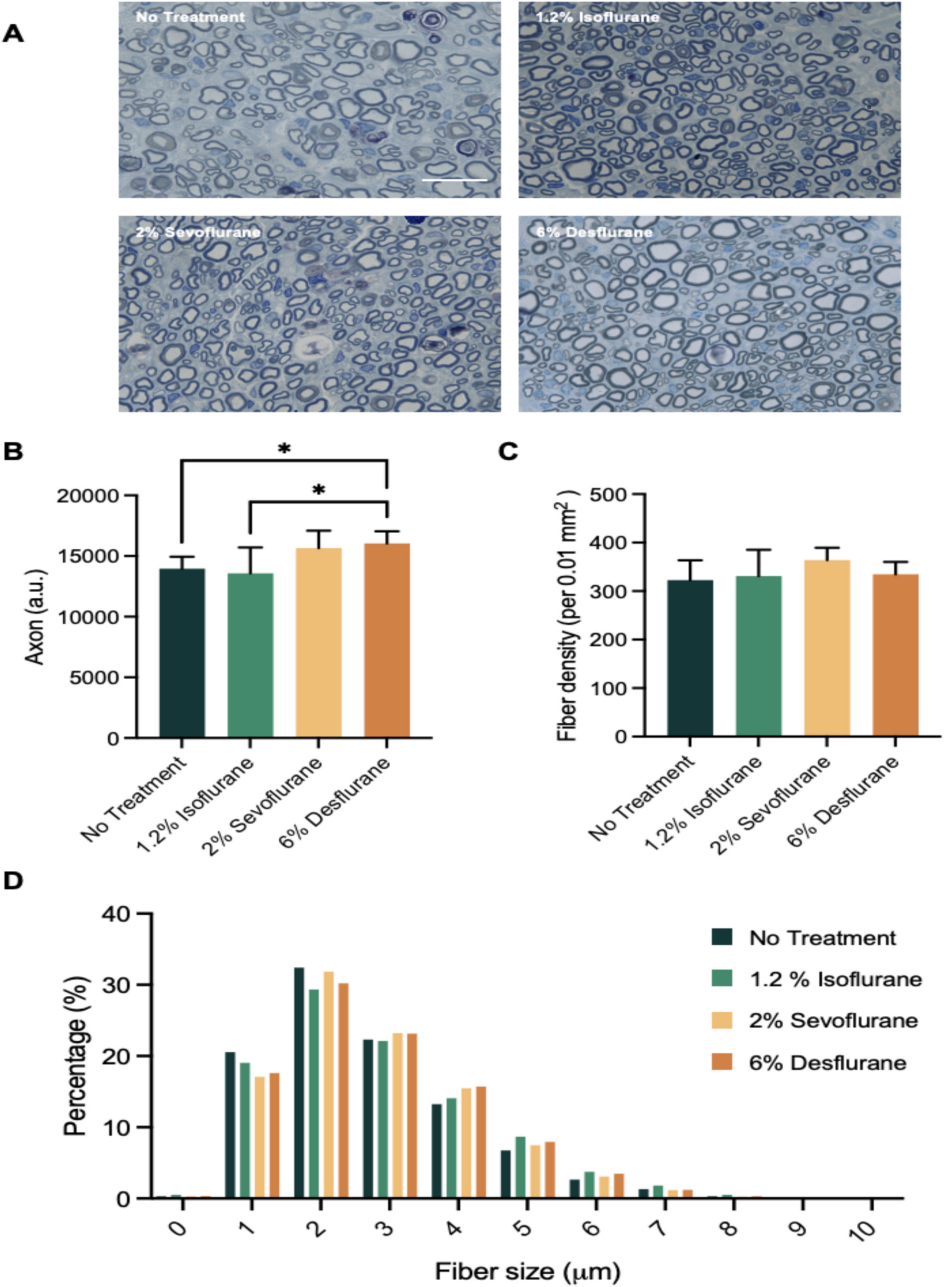
Desflurane promotes axonal regeneration: axonal counts from the sciatic nerve sections at 12 weeks post-surgery. Data are represented as mean ± SD. **(A)** Representative microscopic images of the control and anesthesia treated groups. Scale bar, 30 μm. **(B)** Axonal count, *p* < 0.05, control (No treatment) vs. Desflurane, Isoflurane vs. Desflurane, by Kruskal-Wallis test with Dunn’s multiple comparisons test. **(C)** Fiber density, *p* > 0.05, control (No treatment) vs. Isoflurane, Sevoflurane, Desflurane by Kruskal-Wallis test with Dunn’s multiple comparisons test. **(D)** Distributions of fiber sizes in the control and the different anesthesia treated groups. *N* = 8 individual animals per group. Dark blue, no treatment; Green, isoflurane; Yellow, sevoflurane; Orange, desflurane. *p < 0.05.

**FIGURE 6:**
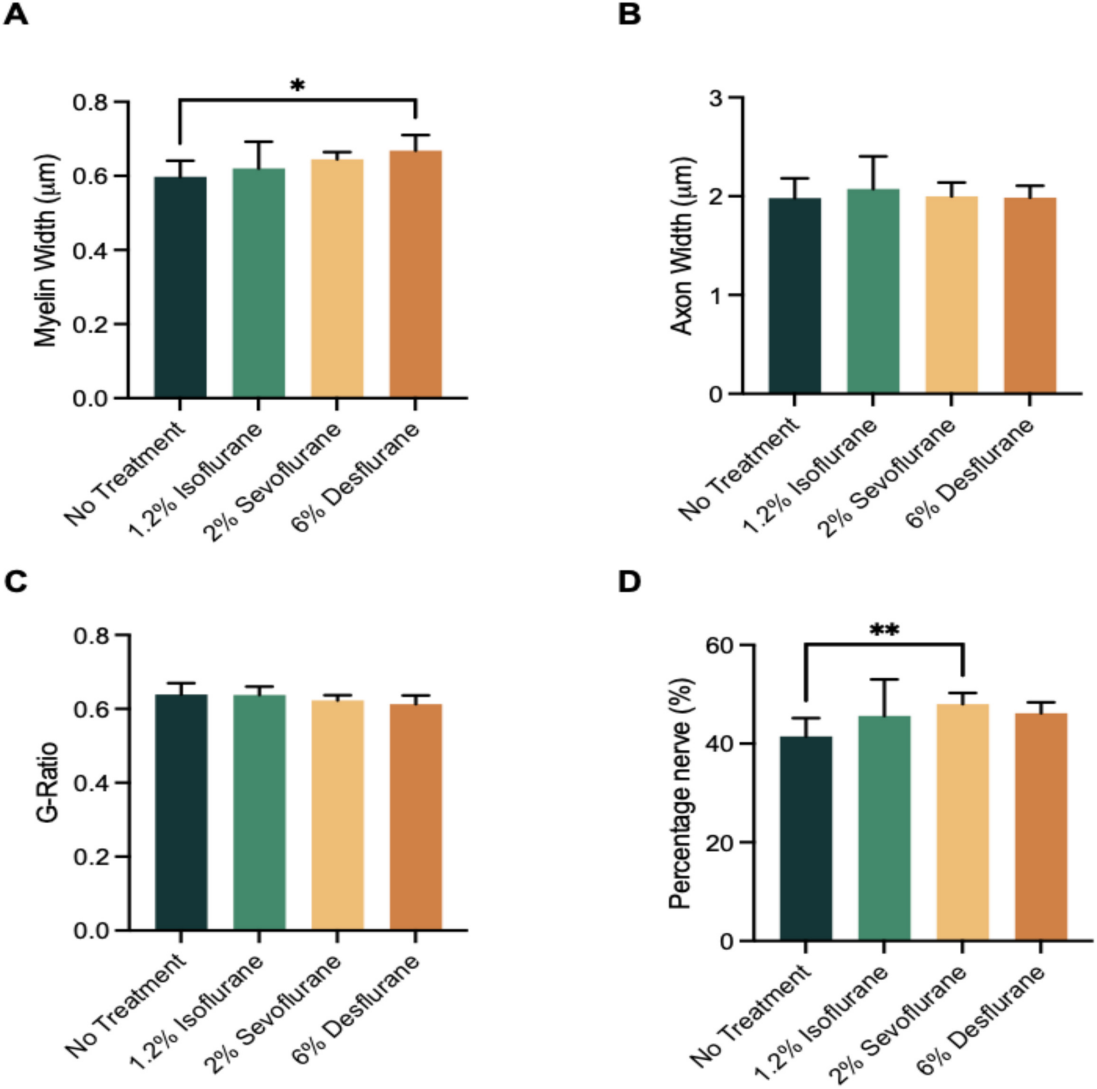
Desflurane improves remyelination: microscopic analysis of axonal structure from the sciatic nerve sections at 12 weeks post-surgery. Data are represented as mean ± SD. **(A)** Myelin width, *p* < 0.05, Desflurane vs. control (No treatment), Isoflurane, Sevoflurane by one way ANOVA with Tukey multiple comparisons test. **(B)** Axon width, *p* > 0.05, control (No treatment) vs. Isoflurane, Sevoflurane, Desflurane by one way ANOVA with Tukey multiple comparisons test. **(C)** G-ratio, *p* > 0.05, control (No treatment) vs. Isoflurane, Sevoflurane, Desflurane by one way ANOVA with Tukey multiple comparisons test. **(D)** Percentage nerve, *p* < 0.05, control (No treatment) vs. sevoflurane, by Welch’s ANOVA with Dunnett’s T3 multiple comparisons test. *N* = 8 individual animals per group. Dark blue, no treatment; Green, isoflurane; Yellow, sevoflurane; Orange, desflurane. *p < 0.05; **p < 0.01.

Figures 5, 6 shows the results of histomorphometric analysis of axonal regeneration. At 12-weeks post-surgery, desflurane showed a significant promotion in axonal regeneration compared to the other groups (Figures 5A,B). The total number of axons in the desflurane group (16041 ± 993) are higher than the control (13959 ± 991), isoflurane (13571 ± 2141), and sevoflurane (15665 ± 1421) groups. The density of nerve fibers, however, shows no significant differences between groups (Control, 322 ± 41 per 0.01 mm^2^; isoflurane, 331 ± 54 per 0.01 mm^2^; sevoflurane, 364 ± 26 per 0.01 mm^2^; desflurane, 335 ± 25 per 0.01 mm^2^) (Figure 5C). Nevertheless, it was noted that the percentage of axonal fibers (larger than 3 μm) was proportionately higher in all the treated groups compared to the control group (Figure 5D).

Figure 6 provides detailed microscopic analysis of the axonal structure. The thickness of myelin sheath was significantly higher in the desflurane group (0.67 ± 0.04 μm) compared to other groups (control, 0.60 ± 0.04 μm; isoflurane, 0.62 ± 0.07 μm; sevoflurane, 0.65 ± 0.02 μm). The axonal width, and the G – ratio (ratio of the inner-to-outer diameter of a myelinated axon) were similar between the control and the treatment groups. The axon sizes in different groups are control, 1.98 ± 0.20 μm; isoflurane, 2.07 ± 0.33 μm; sevoflurane, 2.00 ± 0.14 μm; and desflurane, 1.99 ± 0.12 μm. The G- ratio among the groups are 0.64 ± 0.03, 0.64 ± 0.02, 0.62 ± 0.01, and 0.61 ± 0.02, for control, isoflurane, sevoflurane, and desflurane, respectively. Moreover, an increased percentage of nerve fibers were observed with all the treatment groups (isoflurane, 45.60 ± 7.41%; sevoflurane, 48.02 ± 2.26%; desflurane, 46.17 ± 2.20%) compared to the control group (41.49 ± 3.69%), though a significant difference was noted only between sevoflurane and the control group (Figure 6D).

## Discussion

The main finding in our study is that 1 MAC of desflurane significantly promoted axonal and myelin regeneration at 12 weeks post sciatic cut and repair in a murine model and the similar effect was not observed with the equipotent doses of isoflurane or sevoflurane. This finding is important for several reasons. (1) This suggests that volatile anesthetics may provide neuroprotection in the setting of PNI. These results have significant translational potential given that these anesthetics are already FDA approved, relatively safe, and used in the patients on a regular basis; (2) a differential impact may exist within the volatile anesthetics in providing protection against PNI; and (3) it is possible that volatile anesthetics show a dose dependent protective effect after PNI as we previously showed that 2% isoflurane supported axonal regrowth and myelination resulting in a marked improvement in functional outcomes after PNI but the same effect is not observed with 1.2% isoflurane.

### Differential impact of volatile anesthetics

In the current study we noticed that 1 MAC of sevoflurane did not improve axonal or myelin regeneration compared to 1 MAC of desflurane. Interestingly, this notion is supported by our recent retrospective study, where we noticed that cervical spine injured patients who received desflurane for their peripheral nerve transfer procedures had better motor outcomes (as measured by medical research council scale), compared to the patients who received sevoflurane as their primary anesthetic (Kaleem et al., 2025). This differential effect is also noticed in other neurological conditions such as subarachnoid hemorrhage (SAH), where SAH patients receiving desflurane for their aneurysm repair procedure were associated with reduced incidence of vasospasm and delayed cerebral ischemia compared to the patients who received sevoflurane (Athiraman et al., 2019, 2021). Understanding the effects of different anesthetics is critical as it could guide the optimal anesthetic management for these patients that could possibly improve functional outcomes.

### Dose dependent effects of volatile anesthetics

The 1 MAC of isoflurane (1.2%) in the current study did not support axonal regrowth or remyelination, but 2% isoflurane promoted axonal and myelin regeneration resulting in improved functional outcomes after PNI (Xu et al., 2024). This indicates that the protective effect of isoflurane may be dose dependent. This idea is supported by a few observational studies where the authors showed that higher volatile anesthetic doses were strongly associated with lower odds of postoperative respiratory complications, lower 30-day mortality, lower incidence and severity of postoperative ischemic stroke, and reduced incidence of vasospasm and delayed cerebral ischemia after subarachnoid hemorrhage (Athiraman et al., 2019, 2021; Grabitz et al., 2017; Raub et al., 2021). Interestingly, we observed that 1 MAC isoflurane significantly improved CMAPs, but without increasing the muscle force, indicating that this dose of isoflurane possibly enhances membrane excitability without correcting the downstream effects of improving the force generation (Meola et al., 2009; Wan et al., 2017). Though the reasons are not clear, one of the potential explanations for this finding could be related to the dose or the number of isoflurane exposures. It is also essential to note that though desflurane significantly improved axonal numbers and myelin thickness, it did not improve CMAP. The probable reasons for this finding are (1) desflurane has a greater inhibitory effect on voltage gated sodium channels compared to isoflurane, possibly leading to the reduction in nerve conduction and CMAP, despite preserving the structural integrity of the peripheral nerve (Ouyang et al., 2009); and (2) it is also possible that desflurane by slowing the nerve conduction could have led to desynchronization of axon signals reaching the muscles, eventually reducing the CMAP (Koopman et al., 2025).

It is also important to note that although 1 MAC of desflurane significantly supported axonal and myelin regeneration at 12 weeks post PNI and repair, the improvement in functional outcomes did not reach a statistical significance. The potential causes for this result are (1) a temporal dissociation between nerve regeneration and functional recovery has been shown in the rodent peripheral nerve injury models with increased axonal counts/myelin thickness occurring between 3 and 8 weeks post injury, and the functional recovery occurring by 12–24 weeks post injury (Koopman et al., 2025; Liu et al., 2016). So, it is possible that the functional assessment performed in our study at 12 weeks post PNI could not capture the full functional recovery induced by desflurane; (2) it has been shown that factors other than axonal regeneration could determine functional recovery after PNI (Sobotka and Mu, 2013; Sulaiman and Gordon, 2013; Vannucci et al., 2019). One of the critical factors that determine the functional outcomes after PNI is the neuromuscular junction reinnervation, and this often lags behind the axonal regeneration (Vannucci et al., 2019). It may be that desflurane promoted neuromuscular junction reinnervation at the later time points that were not evaluated in the current study; (3) it is likely that the sample size in our study is not enough to find the potential benefit of desflurane in statistically improving functional outcomes; and (4) it is also possible that the desflurane concentration used in the current study was not sufficient to cause a significant improvement in the functional outcomes and the dose dependent effects of desflurane on improving functional outcomes after PNI should be explored further.

The current study did not explore the underlying mechanistic pathways associated with desflurane’s effect on promoting axonal and myelin regeneration. However, we previously showed that isoflurane, a volatile anesthetic with very close chemical structure to desflurane (Hermann et al., 2000), upregulated some of the genes such as Mtor, Akt1, and Atrn, that are shown to be critical regulators of myelin regeneration (Athiraman and Giri, 2024). So, we speculate that these pathways could have been involved in the desflurane’s effect but this needs to be confirmed in the future studies.

### Limitations and future directions of the study

(1) The sample size in the study may be insufficient to show differences in all the measured outcomes; (2) a longer follow up may be necessary to fully capture the impact of volatile anesthetics on the improvement in functional outcomes after PNI; (3) Isoflurane was utilized as the surgical anesthetic for creating the PNI model in our study and this could have confounded the outcomes in other volatile anesthetic groups such as sevoflurane and desflurane. However, it has been shown that the dose and the duration of isoflurane (less than 20 min) used in our study for the surgery have no protective effects in other neurological disease model (Gaidhani et al., 2017), though this needs to be confirmed in the peripheral nerve injury paradigms; (4) impact of volatile anesthetics on other PNI models such as sciatic crush nerve injury and isograft/allograft are not examined; (5) impact of a commonly used intravenous anesthetic propofol on the peripheral nerve regeneration and functional outcomes has to be examined in the future studies; (6) effects of volatile anesthetics on PNI in female animals has to be explored in the future; (7) a sham group (surgery without sciatic nerve cut and repair) should be incorporated in the future studies to compare along with volatile anesthetics to provide information regarding the baseline nerve function and to delineate the effects of surgical manipulation; and (8) mechanistic pathways underlying volatile anesthetics induced protection after PNI is not examined and should be identified in future studies.

## Conclusion

Our preliminary findings suggest that 1 MAC of desflurane improves axonal growth and remyelination, potentially leading to improved functional outcomes after PNI. Further studies focusing on the optimal dose, and the molecular mechanisms underlying desflurane induced neuroprotection after PNI must be explored.

## Data Availability

The raw data supporting the conclusions of this article will be made available by the authors, without undue reservation.
